# A Fluorescent, Genetically-Encoded Voltage Probe Capable of Resolving Action Potentials

**DOI:** 10.1371/journal.pone.0043454

**Published:** 2012-09-06

**Authors:** Lauren Barnett, Jelena Platisa, Marko Popovic, Vincent A. Pieribone, Thomas Hughes

**Affiliations:** 1 Department of Cell Biology and Neuroscience, Montana State University, Bozeman, Montana, United States of America; 2 The John B. Pierce Laboratory, Inc., New Haven, Connecticut, United States of America; 3 Faculty of Physical Chemistry, University of Belgrade, Belgrade, Serbia; 4 Cellular and Molecular Physiology and Neurobiology, Yale University School of Medicine, New Haven, Connecticut, United States of America; Neuroscience Campus Amsterdam, VU University, The Netherlands

## Abstract

There is a pressing need in neuroscience for genetically-encoded, fluorescent voltage probes that can be targeted to specific neurons and circuits to allow study of neural activity using fluorescent imaging. We created 90 constructs in which the voltage sensing portion (S1–S4) of *Ciona intestinalis* voltage sensitive phosphatase (CiVSP) was fused to circularly permuted eGFP. This led to ElectricPk, a probe that is an order of magnitude faster (taus ∼1–2 ms) than any currently published fluorescent protein-based voltage probe. ElectricPk can follow the rise and fall of neuronal action potentials with a modest decrease in fluorescence intensity (∼0.7% ΔF/F). The probe has a nearly linear fluorescence/membrane potential response to both hyperpolarizing and depolarizing steps. This is the first probe based on CiVSP that captures the rapid movements of the voltage sensor, suggesting that voltage probes designed with circularly permuted fluorescent proteins may have some advantages.

## Introduction

The discovery of the green fluorescent protein (GFP) and its many orthologs (FP) rapidly led to the creation of genetically-encoded, fluorescent biosensors. These probes make it possible to optically record physiologically important signals such as transmembrane potential and intracellular calcium levels. The first voltage sensor was described in 1997 [1], and since then several additional FP-based probes have been described [2]–[6]. The majority of these probes were designed to convert conformational changes in the voltage sensing domains from either an ion channel, or the Ciona intestinalis voltage sensitive phosphatase (CiVSP), into fluorescence changes in a single FP or pairs of FPs. However, to date these probes have lacked the speed to accurately reproduce action potentials with temporal fidelity. While there are detectable, fast components in the response of some of these probes (tau = 8–16 ms), the responses are largely dominated by slow components (tau = >30 ms).

The process of creating better genetically-encoded voltage sensors has lagged behind calcium sensor development [7]. The GCaMP probes [8], [9] are genetically-encoded sensors of calcium ions based on circularly permuted eGFP (cpEGFP) [10], [11]. The crystal structure of GCaMP2 [12] revealed that when the interacting Calmodulin and M13 domains are unbound, a hole appears in the side of the fluorescent protein barrel, likely quenching the fluorophore. Calcium-mediated Calmodulin/M13 interactions in turn occlude this hole and the fluorescence returns. This mechanistic understanding of how GCaMP2 works led directly to the improved GCaMP3 [12], GECO and R-GECO probes [13] with signals that are so robust that the sensors can report changes in intracellular calcium levels from cells deep in living tissues [14], [15].

We sought to leverage recent Ca^++^ probe design principles in our pursuit of improved voltage probes that capture the fastest movements of CiVSP. The design principles used in a previous study that explored circularly permuted FPs as possible reporters in voltage sensors produced no viable probes [16]. However, the development of both Ca^++^ and voltage probes has shown that very small adjustments in the linker between the sensing domain and the fluorescent protein are critical and that a large number of variants need to be rigorously explored. Accordingly, we created and tested 90 different CiVSP::cpEGFP fusions in transiently transfected mammalian cells for the production of fluorescence at the cell membrane and a subset were tested for voltage dependent changes in this fluorescence.

## Results

**Figure 1:**
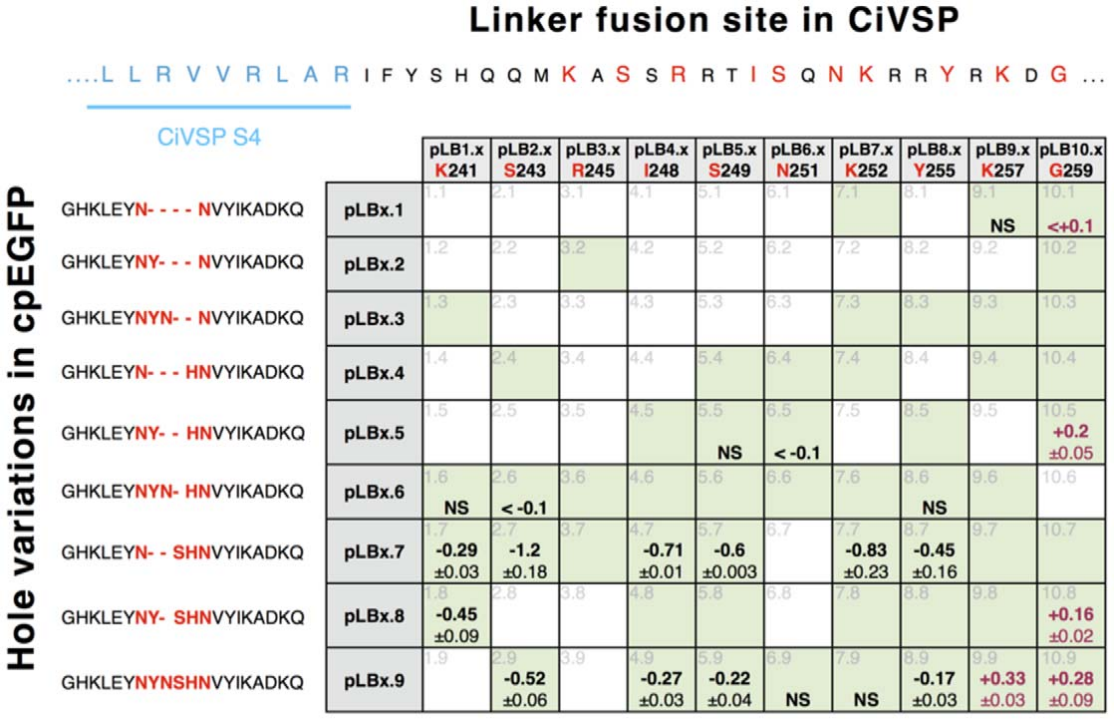
Probe design, expression level and ΔF/ΔV sensitivity of CiVSP::cpEGFP constructs. Two variables were systematically explored: 1) the amino acid position within CiVSP in which the cpEGFP was fused (horizontal axis, e.g.1.x), and 2) the size and position (vertical axis, e.g. x.1) of the hole in the cpEGFP. Gray numbers are construct number. The green shaded boxes within the plot indicate probes that produced fluorescence in HEK 293 cells. Bold numbers in boxes represent average values of peak %ΔF/F ± SEM for +100 mV/200 ms steps from a holding potential of −70 mV; NS = no detectable ΔF/F signal; Red numbers in boxes indicate reversed responses (increase in fluorescence) to depolarizing steps. The response kinetics of constructs 9.1,10.1,5.5,6.5,1.6,2.6,8.6,6.9,7.9 was either non existent or too small to accurately measure. Constructs 7.7 and 8.7 exhibited relatively slow (τ∼69 ms) on and off kinetics. Constructs 10.5,1.7,2.7, 4.7,5.7,1.8,10.8,2.9,4.9,5.9,8.9,9.9,10.9 had on and off rates that were dominated by extremely fast components (τ∼2 ms).

**Figure 2:**
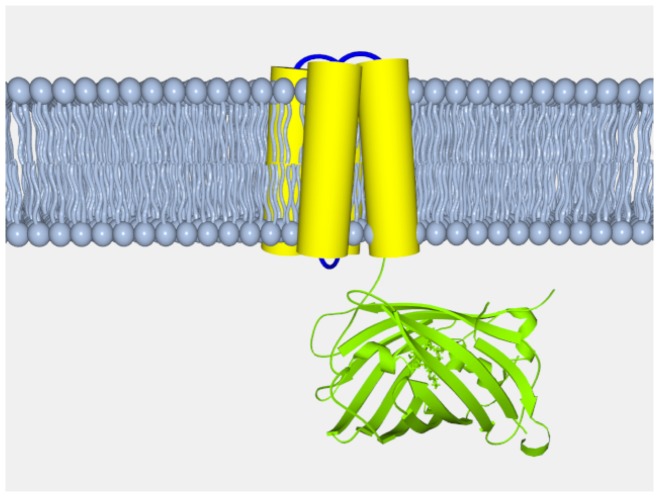
Schematic diagram of CiVSP::cpEGFP based construct design. The S1–4 domain of Ciona intestinalis voltage sensitive phosphatase (yellow) is fused to circularly permuted EGFP (green).

**Figure 3:**
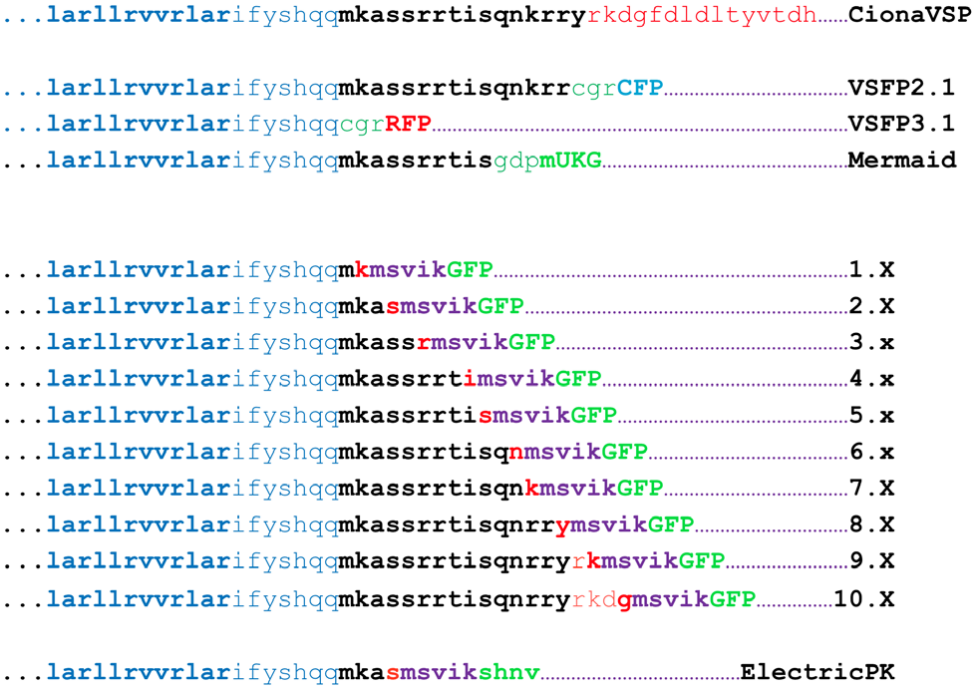
The protein sequence of the various fusion sites of cpEGFP with CiVSP described in this report. From top: CiVSP sequence includes S4 domain (bold cyan), linker domain (cyan and black) and phosphatase domain (red). The fusion sites and linker sequences of VSFP 2.1, 3.1 (17, 18) and Mermaid (4) probes. The ten different fusion sites and linkers described in this report (pLB1.x-10.x; see Figure 1). The fusion site is identified by the last CiVSP amino acid present in the probe (bold red). In all cases, this residue is followed by a five amino acid linker (purple) and the cpEGFP (green). The amino acid at the beginning of the cpEGFP depends on the hole size and position of the cpEGFP (See Figure 1).

In designing the CiVSP::cpEGFP fusions, it was impossible to predict which nearby surface might be available to occlude the hole in cpEGFP, a process that occurs in the calcium-dependent structural changes in GCaMP2 [12]. Most successful CiVSP-based voltage probes involve the fusion of the FP to CiVSP, immediately C-terminal to the S1–4 domains. These fusions truncate the CiVSP, removing the phosphatase domain. The exact fusion site between the CiVSP and the FP plays an important role in voltage sensitivity of the probe. We selected 10 different fusion sites for our probes (Figure 1, 2, 3) all at - or adjacent to - sites used in previous probes. The cpEGFP in GCaMP3 removes amino acids 145 through 148 [13] in the eGFP, but it was unclear whether this would be the right hole size for these constructs, so nine different cpEGFPs were created and fused to each of the CiVSP truncations to produce 90 different constructs for testing (Figure 1, 2).

**Figure 4:**
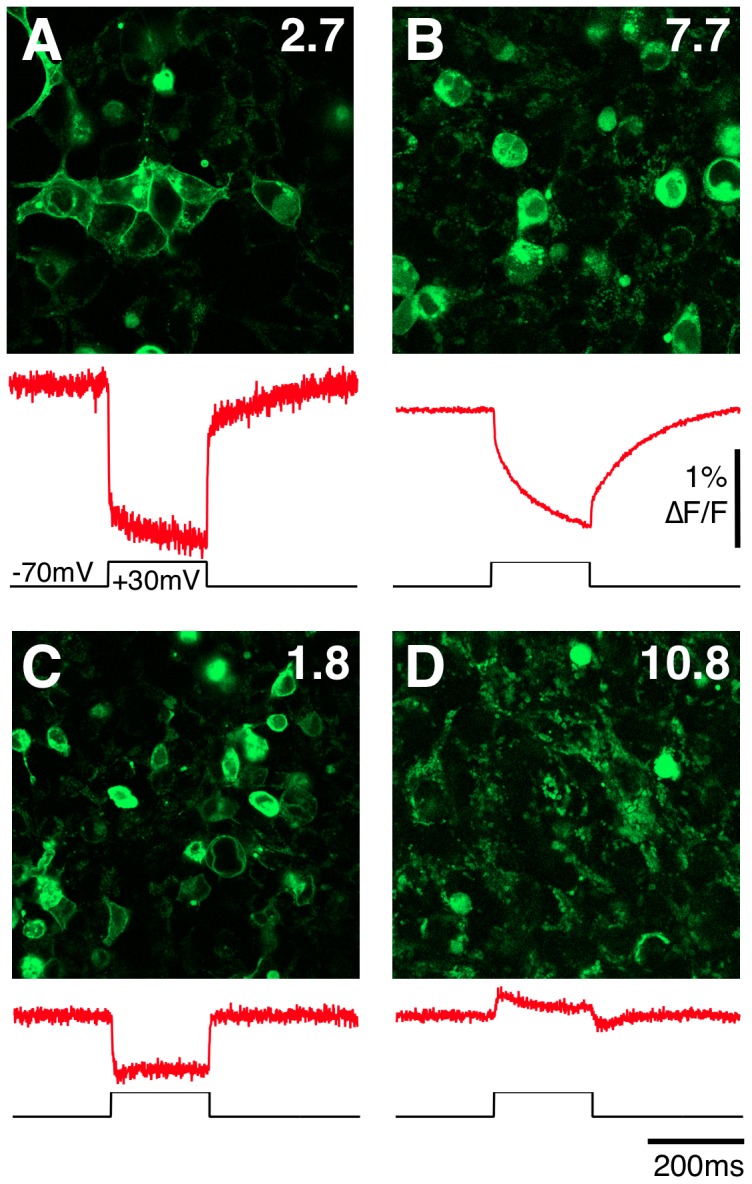
Examples of CiVSP::cpEGFP constructs expression in HEK293 cells and voltage sensitivity. In all cases the upper panel is a confocal image of HEK293 cells transiently expressing the construct and the lower panel is the average fluorescence response (red trace) to ten +100 mV/200 ms voltage steps applied under whole-cell patch clamp configuration. Correction for FP photobleaching has been removed by division of a double exponential fit to the portions of the trace outside the effects of the voltage step. A) The ElectricPk (pLB 2.7) probe is localized both in the membrane and intracellularly. The probe exhibits a rapid decrease in fluorescence with a relatively low signal to noise ratio due to relatively weak basal fluorescence. B) The high expression levels of construct pLB7.7 produces a relatively high signal to noise ratio, however much of the fluorescent protein is localized intracellularly, and its fluorescence response is dominated by a slow component. C) Construct pLB1.8 has mixed membrane and intracellular distribution that produces a small and rapid negative response with a moderate signal to noise ratio. D) pLB10.8 has predominantly intracellular localization and a small positive fluorescence response with a moderate signal to noise ratio.

All constructs were transiently expressed in HEK293 cells and examined for expression level. After 48 hours of expression, 55 of 90 constructs produced fluorescent cells (Figure 1) that were generally of weaker fluorescence and poorer localization (Figure 4) to the plasma membrane when compared to other CiVSP voltage probes [4], [15]–[18]. Comparative analysis of the cellular fluorescence done with confocal microscopy showed variations in the intensity and sub cellular distribution between constructs (Figure 4).

Out of 24 fluorescent constructs that were tested in patch clamp experiments for voltage sensitivity, nine showed negligible (∼0.1% ΔF/F) or no change in fluorescence in response to test voltage steps (+100 mV from a −70 mV holding potential). The remaining constructs exhibited from +0.33% to −1.2% ΔF/F (Figure 1). The largest fluorescence changes were detected in constructs with smaller holes in the FP. In most of the constructs, depolarizing steps provoked a decrease in fluorescence (Figure 4A–C) while five constructs (four at G259 and one at K257; Figure 4D) exhibited increases in fluorescence in response to the same test potentials.

While two (pLB 7.7 and pLB 7.8) constructs exhibited a predominate slow on and off rate (τ∼69 ms; Figure 4B), 13 constructs had on and off rates that were dominated by extremely fast components (τ∼2 ms; Figure 4A and 4C). Our estimate of the maximum response kinetics of these constructs is likely limited by the speed of our voltage clamp (∼1 ms) and the optical sampling rate (2,000 fps) used. The probe with the greatest response magnitude in this series pLB2.7 (−1.2±0.18% ΔF/F;Figure 4A), named ElectricPk, was studied in detail.

**Figure 5:**
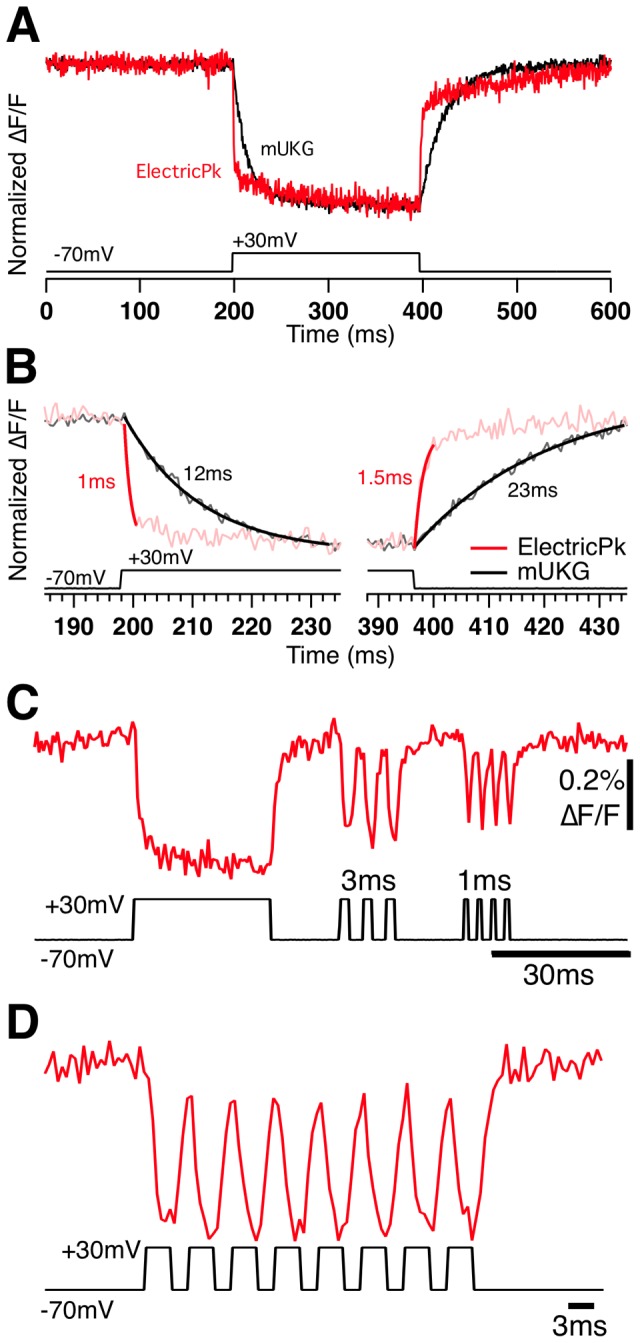
Voltage sensitivity and response kinetics of ElectricPk. A) Voltage-dependent fluorescence sensitivity of an HEK293 cell when transiently expressing ElectricPK (red trace) verses mUKG in Mermaid (upper black trace) tested with voltage steps of +100 mV/200 ms from a −70 mV holding potential (lower black trace). Fluorescence, in all cases, is normalized to initial and peak level. B) Single exponential fits to the dominant on and off response rates of ElectricPk and mUKG of Mermaid in cells tested as in A. C) and D) Response of HEK 293 cell expressing ElectricPk (red trace) to a trains of voltage steps of +100 mV from a −70 mV holding potential with a variable duration; 100 ms, 3 ms and 1 ms (black trace).

ElectricPk exhibited a fast on rate (τ_on_ = 2.24±0.58 ms, n = 8) and off rate (τ_off_ = 2.09±0.74 ms, τ_2off_ = 69.07±20.29 ms; Figure 5A). The on rate of ElectricPK is 5 times faster (Figure 5B) than the CiVSP-based probe Mermaid (τ_on_ = 2.24 ms vs 12 ms, respectively; ref 4) and the off rate is 11 times faster (τ_off_ = 2.09 ms vs. 23 ms, respectively). The speed and response magnitude of this probe make it possible to resolve multiple, high frequency short (1–3 ms) depolarization steps, each of which produce a discrete fluorescence response without a significant accumulating baseline drift (Figure 5C,D).

**Figure 6:**
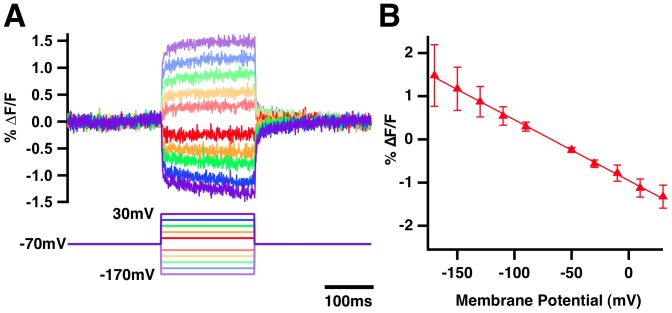
Linear ΔF/ΔV response of ElectricPK. A) Fluorescence response (upper traces) of HEK293 cells transiently expressing ElectricPk to depolarizing and hyperpolarizing voltage steps (−170 to +30 mV from a −70 mV holding potential, lower traces). B) ΔF/ΔV curve of ElectricPk derived from data presented in A.

The fluorescence of ElectricPk exhibits linear relationship to voltage (Figure 6) over a broad range of hyperpolarizing and depolarizing steps (−100 - +200 mV). The voltage/fluorescence relationship became saturated only with large, unphysiologic depolarizing steps (+310 mV; data not shown).

**Figure 7:**
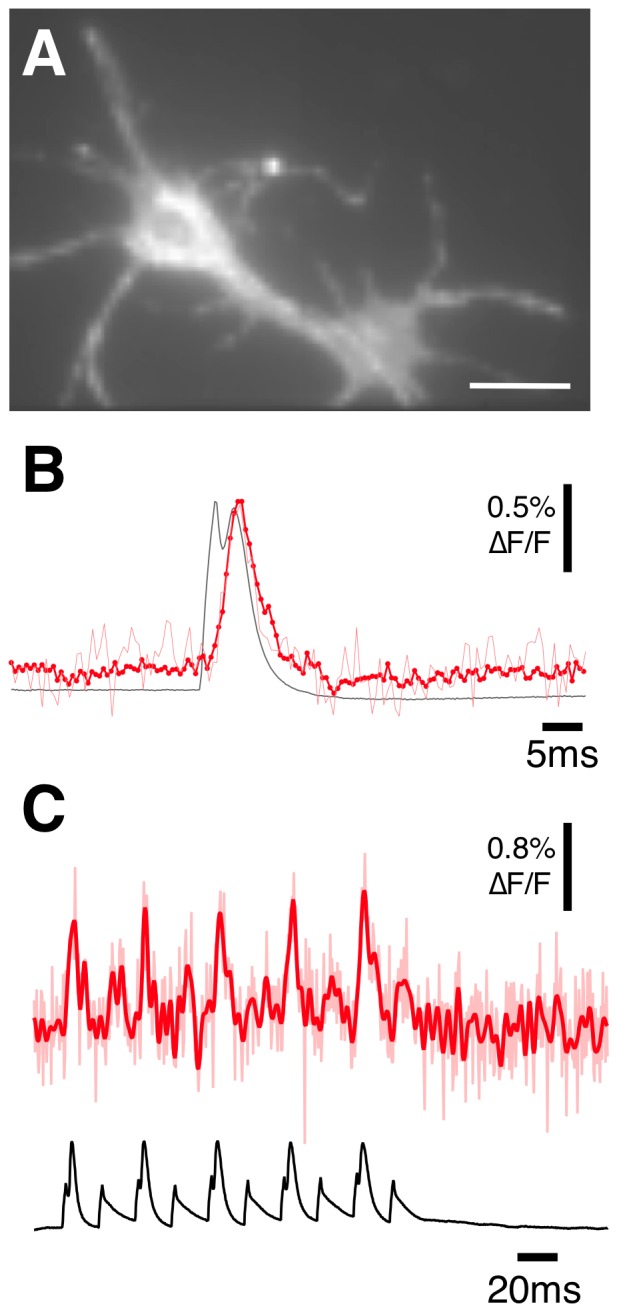
Detection of action potentials in hippocampal neurons in vitro using ElectricPK. A) Wide field image of an in vitro hippocampal neuron expressing ElectricPk. Bar = 15 µm. B) Single (light red trace) and averaged (red trace) optical response to action potentials evoked in the neuron seen in (A) taken using wide field microscopy and a RedShirtImaging NeuroCCD camera. The red trace is an average of 32 action potentials. All responses captured at 2000 fps. C) Fluorescence change (light red trace-unfiltered, red trace-filtered) to a single train of evoked action potentials recorded from an in vitro hippocampal neuron expressing ElectricPk. Lower black trace is the voltage recording made from the patch electrode. All fluorescence traces are bleach subtracted and where indicated, low pass filtered (Bessel) at 350 Hz.

We next expressed ElectricPk in cultured mouse hippocampal neurons. In addition to good membrane localization evident in the cell soma and processes, there were intracellular deposits in some cells, though this was variable (Figure 7A). This may be caused by poorly folded protein, or it could be an artifact caused by abnormal expression levels or the lipofectamine transfection reagents. These intracellular deposits can clearly raise the background fluorescence and lower the signal to noise ratio, so improving on the localization would be beneficial. Fluorescent cells were patch clamped and action potentials generated with brief (2 ms) current injections in current clamp mode. ElectricPk was capable of resolving action potentials with extraordinary temporal fidelity (Figure 7B and Movie S1). The rising and falling phases of the action potential were evident. The signal size was modest (∼0.7% ΔF/F), and varied with expression intensity, yet action potential trains were detectable without trial averaging (Figure 7C).

## Discussion

Early versions of genetically-encoded voltage probes constructed with voltage-gated ion channels [1]–[3] failed to traffic adequately to the plasma membranes of mammalian cells [5]. CiVSP-based probes traffic to the cell membrane more completely and probes created with the CiVSP voltage sensing domain have been more successful in mammalian cells [4], [6], [17]–[19]. The fluorescence changes, however, have been generally slow. The rapid response properties of the probes described here (2 ms) are in striking contrast to the dominant response kinetics (>12 ms) of all other currently published CiVSP-based probes [4], [6], [17]–[19]. The only probes with similar characteristics are the sodium channel-based probe (SPARC; 2) and a probe based on the zebrafish voltage sensitive protein [20]. The SPARC probe was able to capture rapid movements of the VSPs but is less useful due to low signal size and poor membrane targeting [5]. The mechanism by which ElectricPk changes its fluorescence intensity is not clear, but it depends on the circular permutation of eGFP since the fusion of normal eGFP at the same site does not produce viable probes (data not shown).

There appears to be several voltage driven rearrangements in the CiVSP protein. One occurs quite quickly and can be measured as gating charge movement. Others occur more slowly [21], and it is these slow movements that likely drive the changes in fluorescence in many fluorescent voltage probes. The changes in ElectricPk, as well as in the majority of constructs described here, captures only the fast rearrangements in the CiVSP protein as changes in fluorescence. ElectricPk demonstrates that it is possible to capture a voltage dependent movement in CiVSP that previous sensor designs have missed, and to do this in neurons. It appears that the cpEGFP is capturing a fast, voltage-driven movement of CiVSP that both FRET pairs of FPs and single FPs have missed. In addition, the linear F/V response is quite different from all previously published CiVSP-based probes [4], [6], [17]–[19]. This indicates that this probe is likely capturing a different protein rearrangement, one associated with gating charge movements of the S4, which may help to explain its speed.

Previously the Knöpfel laboratory used cpEGFP derived from GCaMP2 to create a few fusions analogous to our constructs [16]. Their VSFP (B) cpEGFP is similar to our pLB1.1 or pLB2.1, and VSFP (C) cpEGFP as well as VSFP (D) cpEGFP are quite like pLB4.1 and pLB7.1, respectively. All of these constructs exhibited very low fluorescence in the initial screen and were not tested for voltage driven changes in fluorescence. Their results, and ours, demonstrate that small changes in the size of the hole in the fluorescent protein and linkage to CiVSP, have profound effects on a probe's response speed and size.

As hyperpolarizing steps cause a linear increase in fluorescence in ElectricPk, some of the constructs that showed no fluorescence during the initial fluorescence screen may have increased fluorescence upon changes in membrane potential. Therefore, we may have missed some probes which exhibited increases in fluorescence in response to voltage steps, as only visibly fluorescent probes were tested for voltage dependent changes in fluorescence using patch clamp. To date, most CiVSP-based voltage sensors have been too slow for following fast neuronal activity. Recently a probe has been developed that makes it possible to detect action potentials, but it too is to slow to clearly resolve the waveform [6]. Tested in cultured neurons, ElectricPk detects action potentials with remarkable time resolution, comparable to what is seen with synthetic, small molecule voltage dyes. This is the first FP-based voltage sensor that could be, with additional adjustments of signal size, used for studies of the origin and propagation action potentials within single cells.

The weak fluorescence of a bacterial rhodopsin has recently been shown to be modulated by transmembrane potential [22]. This approach to genetically-encoded voltage probe development has several potential strengths: large, linear fractional change in fluorescence with voltage; extremely red-shifted excitation and emission spectra and resistance to bleaching. However, the quantum efficiency of fluorescence in these rhodopsins is so low (QY of 0.001 vs 0.65 for GFP) that it is unlikely they will be of much use in optical recordings in vivo. While in vitro conditions are artificially optimized for detecting fluorescence, the low fluorescence of these probes require high intensity (200 mW optical power) laser illumination combined with an EMCCD camera for detection in vitro. These limitations make in vivo use highly improbable with lower levels of expression, more light scattering, and greater sensitivity to phototoxicity. In addition, attempts to produce non-conducting probes have [22], to date, significantly slowed the response kinetics of the probe (<1 ms for wild type vs 45 ms for the non-conducting Arch variant, ArchD95N).

ElectricPk demonstrates that the design principles that have evolved for the creation of better calcium probes can be applied to voltage sensors as well. The present study reconfirms [23] that identifying a viable probe requires a systematic analysis of numerous different fusion location and FP types. While FRET-based sensors offer the theoretical advantage of ratiometric recordings, and should be exquisitely sensitive to small changes in the distance/orientation of the two fluorophores, recent history has shown that sensors designed around a single, circularly permuted FP can produce more robust signals. In this case, ElectricPk captures only the fast movement in the voltage sensing domain of CiVSP that has eluded other sensor designs. While remarkably fast, the signal generated by ElectricPk is relatively small. It is likely that continued rounds of evolution will improve the signal size, much as it has done for calcium sensors.

## Materials and Methods

### Molecular Biology

The region encoding CiVSP (R217Q) was amplified with primers complementary to the translation initiation and a portion of the sequence 3′ of the predicted fourth transmembrane domain. Each reverse primer truncated the CiVSP coding sequence at a different amino acid and appended 15 bp (5 codons) of linker sequence. In turn, the cpEGFP portion of GCaMP3 was amplified with 9 different combinations of 6 primers to create the initial set of fusion components with 15 bp ends identical to the linker sequence at the 5′ end and plasmid at the 3′ end. In-Fusion cloning reactions (Clontech) were used to combine the amplified portions of CiVSP and the fluorescent protein in a CMV expression plasmid adapted for robotic cloning. This plasmid carries a ccdB negative selection cassette flanked by Asc I and Pme I sites such that double digestion removes the selection cassette. In all constructs, the phosphatase domain is not present. ElectricPK is available at Addgene or at the site www.fluorogenetic-voltage-sensors.org.

### Cell culture

The HEK 293 cell line was maintained in the Dulbecco's Modified Eagle Medium (High Glucose) (DMEM) (Invitrogen, NY) supplemented with 8% fetal bovine serum (FBS) (Sigma-Aldrich, MO). This study was carried out in strict accordance with the recommendations in the Guide for the Care and Use of Laboratory Animals of the National Institutes of Health. The protocol was approved by the Pierce Animal Care and Use Committee. Animals were anesthetized with isofluorane prior to removal of embryos. Hippocampal neurons were isolated from E18 mouse embryos and maintained in culture medium containing Neurobasal medium (Invitrogen, NY), Glutamax-I 0.5 mM (Invitrogen, NY) and B-27 supplement (Invitrogen, NY). Cells were plated on No. 1 coverslips coated with poly-D-lysine (Sigma-Aldrich, MO) and kept in an incubator at 37°C with 5% CO2. Transient transfection was accomplished using half of the manufacturer's recommended amount of DNA (2 µg per 35 mm dish) and Lipofectamine 2000 (5 µl) (Invitrogen, NY).

### Electrophysiology

Recordings were performed in a perfused chamber with the bath temperature maintained at 35–37°C and a bath solution containing 150 mM NaCl, 4 mM KCl, 2 mM CaCl_2_, 1 mM MgCl_2_, 5 mM D-glucose, and 5 mM HEPES pH 7.4. We used a 3–5 MΩ glass patch pipettes (capillary tubing with 1.5/0.75 mm OD/ID) that were pulled on a P-97 Flaming/Brown type micropipette puller (Sutter Instrument Company, CA). Pipette solution contained 120 mM K-aspartate, 4 mM NaCl, 4 mM MgCl_2_, 1 mM CaCl_2_, 10 mM EGTA, 3 mM Na_2_ATP and 5 mM HEPES, pH 7.2. Voltage-clamp recordings in the whole-cell configuration were performed using a Patch Clamp PC-505B amplifier (Warner Instruments, CT). A holding potential of −70 mV was kept at all cases.

For hippocampal neuron recordings, action potentials were initiated by 2 ms constant current injection in whole cell patch clamp mode. The pipette solution for neuron recordings contained (in mM): 120 K-gluconate, 3 KCl, 7 NaCl, 4 Mg^2+^-ATP, 0.3 Na-GTP, 20 HEPES and 14 Tris-phosphocreatin, pH adjusted with KOH to pH 7.3 [24].

### Imaging

The whole cell patch-clamped cells were imaged using a Nikon Eclipse TE300 inverted microscope with a 60×1.40 N.A. oil immersion objective lens (Nikon, NY). For HEK 293 cells recordings, we used a 150W Xenon arc lamp (Opti Quip, NY) for episcopic fluorescent illumination using a GFP-3035B filter cube (Semrock Inc., NY) containing a 472/30 nm excitation filter, a 495 nm dichroic mirror and a 520/35 nm emission filter for cpEGFP probes and 445/20 nm excitation filter, a 458 nm dichroic mirror and a 510/84 nm emission filter for Mermaid. For fluorescence recordings in neurons we used 488 nm 50 mW laser illumination (DL488-050, CrystaLaser, NV) that was transmitted to the microscope by a multi-mode fiber coupler (Siskiyou, OR), a quartz light guide and an Achromatic EPI-Fluorescence Condenser (Till Photonics, NY). The fluorescence image was demagnified by an Optem® zoom system A45699 (Qioptiq LINOS, Inc, NY) and projected onto the 80×80 pixel chip of a NeuroCCD-SM camera (RedShirtImaging, LLC, GA) controlled by NeuroPlex software (RedShirtImaging, LLC, GA). The images were recorded at a frame rate of 2 kHz. The measured fluorescence was the average intensity across the entire cell.

Confocal images were obtained with a Olympus Fluoview FV1000 (Olympus, Japan) confocal laser scanning microscope using an Olympus LUMFL 60×/1.10 W objective. For chromophore excitation 488 nm wavelength Ar-laser was used. We used EGFP filter set with excitation filter 488BP (480–495 nm), beam splitter 505 nm and for emission filter BP515 (505–525 nm). For image acquisition and processing FV10-ASV confocal software was used.

### Data Analysis

Data were analyzed with custom programs written in LabView (National Instruments Inc.,TX) and Igor (Wavemetrics, OR). Bleaching was corrected by dividing the signal by a double exponential curve fitted to the portion of the trace that does not contain a voltage step. Data are presented as percent change in ΔF/F.

## Supporting Information

Movie S1 **The movie illustrates the optical response of an action potential (AP) in a pyramidal neuron from a hippocampal cell culture expressing the ElectricPK probe.** The value of each pixel is the spike-triggered average value (over 43 action potentials) aligned to the peak of the action potential in the somatic whole cell recording. First, a low pass temporal filter (Gaussian at 200 Hz) is applied to the averaged data. Next, five iterations of spatial filtering in which each data pixel is averaged within a window of the 3×3 array of pixels surrounding it is applied. The color code in each pixel is scaled so that blue is the most hyperpolarized membrane potential, red is the most depolarized membrane potential (e.g. AP peak) and yellow is the half-way point of the spike. All data processing (averaging, bleach correction, filtering) was done according to the procedure described in Popovic et al. [19].
(MOV)
